# Megalocytivirus and Other Members of the Family *Iridoviridae* in Finfish: A Review of the Etiology, Epidemiology, Diagnosis, Prevention and Control

**DOI:** 10.3390/v15061359

**Published:** 2023-06-12

**Authors:** Pan Qin, Hetron Mweemba Munang’andu, Cheng Xu, Jianjun Xie

**Affiliations:** 1 Key Laboratory of Marine Biotechnology of Fujian Province, College of Marine Sciences, Fujian Agriculture and Forestry University, Fuzhou 350002, China; panqin@fafu.edu.cn; 2 Faculty of Biosciences and Aquaculture, Nord University, 8026 Bodø, Norway; hetron.m.munangandu@nord.no; 3 Department of Paraclinical Sciences, Faculty of Veterinary Medicine, Norwegian University of Life Sciences, 1433 Ås, Norway; cheng.xu@nmbu.no; 4 Key Laboratory of Mariculture and Enhancement of Zhejiang Province, Marine Fisheries Research Institute of Zhejiang, Zhoushan 316100, China

**Keywords:** megalocytiviruses, ranaviruses, lymphocystiviruses, vaccines, biosecurity, etiology, epidemiology, diagnosis, prevention, control

## Abstract

Aquaculture has expanded to become the fastest growing food-producing sector in the world. However, its expansion has come under threat due to an increase in diseases caused by pathogens such as iridoviruses commonly found in aquatic environments used for fish farming. Of the seven members belonging to the family *Iridoviridae*, the three genera causing diseases in fish comprise ranaviruses, lymphocystiviruses and megalocytiviruses. These three genera are serious impediments to the expansion of global aquaculture because of their tropism for a wide range of farmed-fish species in which they cause high mortality. As economic losses caused by these iridoviruses in aquaculture continue to rise, the urgent need for effective control strategies increases. As a consequence, these viruses have attracted a lot of research interest in recent years. The functional role of some of the genes that form the structure of iridoviruses has not been elucidated. There is a lack of information on the predisposing factors leading to iridovirus infections in fish, an absence of information on the risk factors leading to disease outbreaks, and a lack of data on the chemical and physical properties of iridoviruses needed for the implementation of biosecurity control measures. Thus, the synopsis put forth herein provides an update of knowledge gathered from studies carried out so far aimed at addressing the aforesaid informational gaps. In summary, this review provides an update on the etiology of different iridoviruses infecting finfish and epidemiological factors leading to the occurrence of disease outbreaks. In addition, the review provides an update on the cell lines developed for virus isolation and culture, the diagnostic tools used for virus detection and characterization, the current advances in vaccine development and the use of biosecurity in the control of iridoviruses in aquaculture. Overall, we envision that the information put forth in this review will contribute to developing effective control strategies against iridovirus infections in aquaculture.

## 1. Introduction

Iridoviruses are important pathogens of ectothermic vertebrates, causing a large number of deaths in fish, frogs, newts, and many other wild and cultured lower vertebrates in many regions of the world [1]. Based on the virus particle size, host range, GC content of the virus genome, major capsid protein (MCP) gene similarity, clinical disease and other main characteristics, the family *Iridoviridae* is divided into seven genera [2,3,4], namely *Ranavirus*, *Megalocytivirus*, *Lymphocystivirus*, *Iridovirus*, *Chloriridovirus*, and the newly discovered *Decapodiridovirus*, and *Daphniairidovirus* (https://ictv.global/taxonomy, accessed on 4 May 2023). Among them, *Ranavirus*, *Megalocytivirus*, and *Lymphocystivirus* mainly infect vertebrates, especially ectothermic vertebrates that live in humid or aquatic environments, such as fish, amphibians, and reptiles [5,6]. All three above mentioned genera of *Iridoviridae* have been found to cause disease in fish and they are now widely considered as a great threat to both wild fish populations and farmed fish. They infect a wide host range of fish species including top farmed commercial fish such as tilapia (*Oreochromis niloticus*), seabream (*Pagrus major*), largemouth bass (*Micropterus salmoides*), grouper and salmonids. They are ubiquitously found in different aquatic environments for which their presence in aquaculture is bound to cause high economic losses to the fish farming industry globally. Thus, the objective of this synopsis is to bring into perspective the current state of knowledge on the etiology, diagnosis, epidemiological factors linked to the occurrence of outbreaks as well as the measures used for the control and prevention of iridovirus diseases in farmed fish.

## 2. Historical Perspective

### 2.1. Genus Ranavirus

Largemouth bass virus (LMBV), a member of the genus *Ranavirus*, was first isolated from largemouth bass in a sporadic to epidemic manner in Lake Weir, Florida, US, in 1991 [7,8]. The nomenclature of this pathogen was given years later when an incident of death in salmon involving more than 1000 adults between 2 and 6 kg was investigated at the Sandy Cooper Reservoir in South Carolina, USA in 1995 [9]. Subsequently, LMBV was detected in 17 states in the US [10]. In addition, the viruses referred to as Doctor fish virus (DFV) and Guppy virus-6 (GV6) that were isolated from apparently healthy ornamental fish were found to have the same lineage as LMBV. Now, the three viruses mentioned above all belong to the Santee-Cooper ranavirus species [11,12]. Overall, various studies reported that LMBV has a much narrower host range primarily infecting centrarchids [13,14]

Before the isolation of epizootic hematopoietic necrosis virus (EHNV), in 1985, in redfin perch in Australia, it was not known that EHNV, which also belongs to the *Ranavirus* genus, would cause systemic infection and death in finfish [15]. In fact, EHNV was the first iridovirus reported related to epizootic mortality in vertebrates [15,16,17,18,19]. The first epidemic of EHNV was in freshwater reservoirs in central Victoria (VIC), to be more specific, Lake Nillahcootie and Lake Mokoan in the Brock River Basin, Australia [20]. Several wild perch populations (*Perca fluviatilis*) in northeastern Victoria were infected by EHNV between 1984 and 1986, raising serious concerns because of its capacity to cause massive fish die-off [15,21]. The pathology caused by EHNV is characterized by multifocal necrosis of the renal hematopoietic interstitium, liver and spleen in redfin perch. The foci of necrosis are often centered on blood vessels and include necrosis of endothelial cells [22]. Another closely related member of the genus *Ranavirus*, namely, the European catfish virus (ECV), was isolated from sheatfish (*Silurus glanis*) fry in a recirculating aquaculture facility in Germany, in 1988 [23]. Despite having the same lineage, EHNV and ECV can be distinguished by simple and rapid molecular detection [24]. Between these two species of *Ranavirus*, EHNV is limited to Australia and ECV is restricted to continental Europe [20].

In Singapore, recurrent diseases causing high cumulative mortality and no premonitory clinical symptoms except drowsiness and anorexia were reported in brown-spotted grouper (*Epinephelus tauvina*), in 1992 [25], and Malabar grouper (*Epinephelus malabaricus*), in 1998 [26]. The etiological agent was later identified as Singapore grouper iridovirus (SGIV), which is a member of the genus *Ranavirus* and the main pathogen of marine cultured grouper, causing serious systemic diseases in grouper fry, with a mortality rate of more than 90% [27,28]. SGIV was first isolated in 1998, which caused huge economic losses to grouper culture in many Southeast Asian countries [25,28,29]. A related grouper iridovirus (GIV) was later identified as a rana-like virus based on the whole genome sequence in further outbreaks [30].

### 2.2. Megalocytivirus

*Megalocytivirus* represents one of the most important pathogens causing high mortality in many finfish species. As pointed out by different scientists [31,32], *Megalocytiviruses* have been reported to mostly cause disease in fish species belonging to the *Perciformes*, *Pleuronectiformes* and *Tetraodontiformes*, indicating that they have a wide host range that includes several farmed-fish species in aquaculture. As such, more than 30 marine and freshwater fish species from Japan, the South China Sea, and several Southeast Asian countries have been reported to be susceptible to infections by *Megalocytiviruses* [33]. Kurita and Nakajima reported more than 50 marine and freshwater fish species as susceptible to infection by *Megalocytiviruses* [6]. High mortality reaching 100% have been reported both in natural and experimental infections. Based on phylogenetic similarities of the major capsid protein (MCP) and adenosine triphosphatase (ATPase) genes, *Megalocytivirus* is divided into three genotypes, namely red sea bream iridovirus (RSIV), infectious spleen and kidney necrosis virus (ISKNV) and turbot reddish body iridovirus (TRBIV) [6,34]. RSIV and ISKNV can be further divided into two subtypes based on phylogeny. RSIV subtype 1 is closely related to the ehime-1 strain isolated from red sea bream (*Pagrus major*), in 1990 [35]. This virus, however, is rarely reported in recent epizootics, even in Japan [36]. RSIV subtype 2 is closely associated with rock bream iridovirus (RBIV), a representative pandemic strain in marine aquatic farms [37,38]. The ISKNV subtype 1 was first isolated from mandarin fish (*Siniperca chuatsi*) in 1998 [39]. It is found mainly in freshwater fish [40]. Phylogenetic analysis revealed that some viruses, isolated from 2006 through to 2011, from Bangaii cardinal fish (*Pterapogon kauderni*) and marble sleepy goby (*Oxyeleotris marmorata*) belonged to genotype II [41,42]. In 2004, a fish disease causing high mortality and severe tissue damage in turbot was reported in China. The virus was then classified as an iridovirus and named as turbot reddish body iridovirus (TRBIV) [43]. TRBIV is closely related to flounder iridovirus (FLIV) variants [44]. TRBIV isolates were considered to be restricted to East Asia and have been classified as the TRBIV genotype 1 [45]. Currently, there are TRBIV clade II genotypes found in freshwater ornamental fish [45,46] and marine-reared rock bream [47]. In recent years, outbreaks of large yellow croaker iridovirus (LYCIV) have been increasingly reported in China. The virus was first isolated from cultured large yellow croakers in China in 2003 [48]. The virus mainly infected large yellow croaker juveniles, and the virus particles were present in the spleen and kidney of diseased fish [48]. As the virus failed to grow in commonly used cell lines, the pathogenicity and biological characteristics of LYCIV remain to be elucidated. Some researchers argue that LYCIV is the causative agent of “white gill disease”, which caused mass mortality in cage-reared large yellow croakers. However, others consider that this virus is only a co-infectious agent which causes disease in large yellow croakers with other pathogens. In 2020, Wang et al. isolated and identified a novel iridovirus, named LYCIV-ZS-2020, from cage-cultured large yellow croaker and conducted the artificial infection trial [49]. The cumulative mortality of the artificial infection trial was much lower than that observed in natural infection [49], indicating the pathogenicity of LYCIV to large yellow croaker is limited, and higher mortality might be caused by co-infections with unknown pathogens in seawater.

### 2.3. Lymphocystivirus

Lymphocystis disease has been reported in over 125 different fish species from 34 different fish families [50] showing its potential to have a devastating impact in aquaculture. After Lowe first discovered lymphocystis disease in flatfish in 1874 [51], Walker then observed the pathogen causing such disease, described its morphology and structure under electron microscope in 1962, and named it lymphocystis disease virus 1 (LCDV-1) [52]. Currently, the genus *Lymphocystis disease virus* includes three virus species, namely lymphocystis disease viruses 1, 2, and 3, whose complete genome sequences have been determined. The whole genome sequence of LCDV-1 was later obtained in 1997 [53]. The International Committee on Taxonomy of Viruses (ICTV) currently recognizes LCDV-1 and also tentative species including lymphocystis disease virus 2 (LCDV-2) and lymphocystis disease virus China (LCDV-C) [54]. In 2004, Zhang et al. reported the complete genome sequence of LCDV-C [55] whereas Kawato et al., in 2021, reported the genome of LCDV 2 LCDV-JP_Oita_2018, which was isolated from a Japanese flounder (*Paralichthys olivaceus*) [56]. However, Doszpoly et al. [57] recently reported another lymphocystivirus detected in white mouth croaker (*Micropogonias furnieri*) and proposed that it be classified as LCDV-4, but the name of the virus species has not yet been accepted by the ICTV.

## 3. Etiology

### 3.1. Structure and Genome Organization of Fish Iridoviruses

*Iridoviridae* is a family of large dsDNA viruses with icosahedral symmetry and a diameter of 120–300 nm, and some fish lymphocystis disease viruses even reach 380 nm in diameter [58,59]. The virus particles are mainly composed of the capsid, intermediate lipid layer and core body, and some iridoviruses released from budding have an outer membrane outside the capsid protein [58,59]. The capsid of iridoviruses constructed by the major capsid protein (MCP) has icosahedral symmetry with T = 147 triangulation numbers. The MCP contains 40% of the structural proteins, together with about 36 additional polypeptides, participating in viral particle formation [1,60]. Fibers with unknown function exist on the surface of the capsid, and the inner surface of the capsid is surrounded by an inner lipid membrane and is bound with additional structural proteins. In budded virions, the outer membrane consisting of lipids and glycoproteins is obtained during sprouting from the host cell membrane. This adventitia is not formed in virions released during cell lysis, and it is not necessary for infectivity [1,61].

At the center of the core is the linear double-stranded DNA genome, which is packaged into nucleoprotein filaments together with related proteins. Iridoviruses have a huge, 105–200 kilobase pairs, a highly methylated genome, with a circular arrangement and terminal redundancy. The genome encodes about 100 viral proteins, depending on the genus, most of which have unknown functions and are exclusive to the virus family [61]. There are 26 core genes that are conserved in the family; the diversity of other genes reflects the universality of the host and environment [62]. Both ends of the genomic double-stranded DNA molecules of *Ranavirus*, *Lymphocystivirus* and *Iridovirus* have a repetitious gene sequence, which is known as “terminal redundancy”. Another DNA structure shared by iridoviruses is circular permutation, which is a different terminal circular arrangement at both ends of different molecules [5,63].

### 3.2. Core Gene and Potential Function

Genome sequence comparison shows that there are significant differences between megalocytiviruses, lymphocystiviruses, and ranaviruses, given that the GC content of lymphocystiviruses varies between 27 and 29% is considerably lower than that of ranaviruses and megalocytiviruses that varies between 49 and 55% (Table 1) [64]. Compared with other viruses, the iridoviruses have a huge genome and many genes encoded. Although there are more than 20 genome sequences of *Iridoviridae* that have been sequenced, most open reading frames (ORFs) have not been verified at the transcription and translation level. Through the unified standard annotation and comparative analysis of the sequenced iridovirus genome sequence, it was found that there are 26 relatively conservative and important core genes in the *Iridoviridae* family genome [65]. Except for 2 core genes with unknown function, the remaining 24 core genes have predicted functions that can be categorized into (1) DNA replication and repair proteins; (2) nucleotide metabolism-related proteins; (3) transcription and translation regulation proteins; (4) structural proteins [66].

Among the above mentioned core genes, the major capsid protein (MCP) gene is the one that has been most widely studied and commonly used in iridovirus detection. The MCP of iridoviruses has a relative molecular mass of about 50 kDa. It accounts for 90% of the soluble protein in the virus leading to the production of neutralizing antibodies against the virus in the host. Under most circumstances, the MCP genes of different iridoviruses have different lengths and they encode a different number of amino acids [65]. As a representative strain of the genus *Ranavirus*, the MCP gene of frog virus 3 (FV3) has a total length of 1392 bp and encodes 463 amino acids [67]. The MCP gene of LCDV virus of the genus *Lymphocystivirus*, on the other hand, is 1380 bp in length and encodes 459 amino acids; the ISKNV virus of the *Megalocytivirus* has a full-length MCP gene of 1362 bp, and the protein it encodes has 453 amino acids. Nevertheless, there are cases where different iridoviruses have equal lengths of the MCP gene sequence and the same number of encoded amino acids. For example, although RSIV and ISKNV are two genotypes of the genus *Megalocytivirus*, their MCP genes are both 1362 bp in length and encode 453 aa. Because the homology of the MCP gene sequence and the encoded amino acid sequence reflect the genetic relationship between the different iridescent viruses, the MCP gene is regarded as the basis for studying the molecular evolution of iridoviruses. The analysis of the MCP gene sequence of viruses derived from *Lymphocystivirus*, *Ranavirus* and *Megalocytivirus* revealed that although the host ranges of these viruses are different, their MCP genes still have highly conserved domains [54,68,69].

## 4. Diagnostic Assays

### 4.1. Clinical Signs and Pathology

Fish infected with iridoviruses usually develop the following symptoms: dark body color, abnormal swimming behavior, lethargy, gill hyperemia or bleeding. In severe cases, the diseased fish may have protruding eyeballs and overgrowth of the gills. Necropsy usually shows obvious symptoms of anemia, such as pale liver, spleen and kidney enlargement, accompanied by bleeding spots. However, the same virus can also sometimes elicit quite different clinical symptoms in the same fish species. For example, Kim et al. examined turbot (*Scophthalmus maximus*) after infection with the turbot reddish body iridovirus; the bodies of diseased fish turned pale, with protruded eyes and swollen abdomens [70], whereas in the study of Shi et al., the symptoms of *turbot Scophthalmus maximus* infected with the turbot reddish body iridovirus were pale gills with local hemorrhages on the fins and fin base, and severe hemorrhage in muscle and skin [43]. Despite this, splenomegaly is the most common and critical manifestation of diseases caused by iridoviruses. Therefore, for diagnosis, the spleen is the most important target organ used for histopathological examination rather than other organs such as the kidney, gill, liver, heart and intestine.

### 4.2. Giemsa Staining

The examination of histological sections by Giemsa staining of the spleen from diseased fish reveals abnormally enlarged cells. Geimsa staining can be used to identify characteristic eosinophilic inclusions and associated pathological lesions in the spleen of infected fish [71]. In addition, Giemsa staining of erythrocytes can be used to identify inclusion bodies and vacuole formation in the cytoplasm [32,72].

### 4.3. Virus Isolation and Cell Culture

Successful isolation, culture and identification of viruses from infected organs is the “gold standard” for the diagnosis of iridovirus infections. However, this can sometimes be a daunting task. In the case of iridoviruses, the challenge comes from difficulties in finding sensitive cells for virus cultivation. So far, only a few sensitive cells are reported to be suitable for the culture of iridovirus. As for *Ranaviruses*, fish cell lines such as fathead minnow (FHM), bluegill fry (BF-2) and Chinook salmon embryo (CHSE-214) have been used to isolate EHNV [15,73] (Table 2). The isolation and identification of ECV can be performed in FHM, BF-2, Epithelioma papulosum cyprini (EPC) cells, and channel catfish ovary (CCO) cells, with BF-2 cells being the best option [23,73] (Table 2). Several of the abovementioned cell lines can also be used for the isolation of LMBV, including BF-2, FHM and EPC cells [74]. Cell lines suitable for SGIV and GIV isolation are also shown in Table 2. SGIV has been isolated from *Epinephelus akaara* grouper kidney (EAGK) cells, a cell line derived from the kidney of grouper [75]. As for *Megalocytiviruses,* RSIV can be grown in several cell lines such as BF-2, grunt fin (GF), and red-spotted grouper embryo (KRE-3) (Table 2). The GF cell line is recommended by the OIE for isolating RSIV and ISKNV, but it is difficult to cultivate these two viruses with cell lines derived from freshwater fish. Cell lines used for the culture of other members of the genus *Megalocytivirus* are shown in Table 2. Cell lines used for the culture of *Lymphocystiviruses* include the BF-2, CHSE-214, EPC, *Sparus aurata* fibroblast (SAF-1), turbot (*Scophthalmus maximus*) kidney (TK), brown-marbled grouper fin cell line (bmGF-1) and *Cynoglossus semilaevis* gonad cell (CSGC) (Table 2). As shown in Table 2, some cell lines, such as BF-2, FHM and EPC cells, can be used to isolate different iridoviruses belonging to different genera.

### 4.4. Molecular Diagnostic Methods

As a diagnostic method at the molecular level, PCR detection is simple, fast, sensitive, and highly accurate. It has been widely used in the detection of various pathogens, including iridoviruses. The sequence similarity of the MCP genes of the different *Iridoviridae* genera is about 50%. The similarity between the members of the genus *Ranavirus* can reach 75%. Therefore, primers whose design is based on the conservative sequence of the MCP gene are commonly used to detect iridoviruses from diseased aquatic animals in various places [110]. PCR amplification of the frog virus MCP gene and the immediate early protein A gene identified the frog virus as the pathogen causing tadpole death in cultured frogs [111]. Marsh et al. used PCR to amplify the MCP sequence and successfully distinguished different members of the *Ranavirus* found in Australia, Europe and the United States based on the differences in the restriction endonuclease patterns of specific PCR products [24]. With the MCP gene as the target gene, conventional PCR and TaqMan quantitative PCR detection methods were established to detect FV3-like viruses from Terrapene Carolina. The results showed that both methods could specifically detect FV3-like viruses. They also found that the sensitivity of TaqMan quantitative PCR was 1000 times higher than that of conventional PCR [112,113]. It was also reported that RSIV and dwarf gourami iridovirus could be detected by using the conserved sequence target of the MCP gene [114]. RSIV, along with ISKNV, can also be detected by viral PstI restriction fragment-targeted conventional PCR, which is the molecular detection method recommended by OIE [115]. The disadvantage of this method is that sequence mismatch may lead to false negative results. For example, a Chinese RSIV strain isolated by Jeon et al. could not be detected using the OIE recommended method due to sequence mismatch [116]. Both conventional and real-time PCR can be used to detect LMBV with good specificity [117]. For LCDV, the conventional PCR method had good sensitivity in the diagnosis of LCDV-1 subclinical infection [118] while multiplex PCR was able to detect multiple LCDV genotypes [119].

As a low-cost alternative to PCR, loop-mediated isothermal amplification (LAMP) analysis has also been used for the rapid detection of iridoviruses such as ISKNV, TRBIV, and some LCDVs [120,121,122,123]. Further, the restriction fragment length polymorphism (RFLP) method was used for detection of LMBV [8].

### 4.5. Immunoassays

Immunoassays used for the detection of iridoviruses include the enzyme-linked immunosorbent assay (ELISA) for the detection of EHNV [124], [16], SGIV [125], LMBV [126], RSIV [127] and other iridoviruses [128,129]. Currently, ELISA is becoming more quantitative and accurate, reaching detection limits as low as 10^3^ PFU/mL due to ongoing technological refinements [130,131]. Immunohistochemistry (IHC) staining has been developed for the detection of viral antigens in infected tissues for viruses such as LMBV [132], RISV [133], TGIV [134]. Iridovirus isolated from the marine giant sea perch causes infection, EHNV [16,135], European sheatfish virus (ESV) [136], ECV [136], pike–perch iridovirus (PPIV) [136], New Zealand eel virus (NZeelV) [136] and ISKNV [137]. The location of viruses linked to histopathological tissue damage can also be observed by IHC using monoclonal antibodies targeting the infecting iridovirus [138]. In addition, immunofluorescent antibody tests (IFAT) have also been developed for the detection of different iridoviruses including LMBV [132], RISV [139], ECV [140], and EHNV [16].

### 4.6. In Situ Hybridization and Transmission Electron Microscopy

An important technique for visualizing virus localization is the use of in situ hybridization (ISH) that uses molecular probes to detect specific viral nucleic acid sequences in fixed tissues. For example, Huang et al. [141] developed an ISH staining technique able to detect SGIV nucleic acids in the formalin-fixed tissue of grouper (*Epinephelus malabaricus*). Similarly, Glen et al. [142] used a transmission electron microscope (TEM) for the detection of erythrocytic necrosis virus (ENV), whereas Davies et al. [143] used acridine orange staining for the indirect detection of ENV, the green fluorescence of which was only seen when bound to the ENV double stranded DNA. Likewise, Haytt et al. [16] used immunoelectron microscopy to detect EHNV in redfin perch (*Perca fluviatilis*) and rainbow trout.

### 4.7. Other Diagnostic Methods

Apart from the above mentioned diagnostic methods currently used in the diagnosis of different iridoviruses in finfish, other novel approaches are being developed. For example, Qin et al. [144] developed a sensitive and accurate flow cytometry (FCM) method to detect and quantify the percentage of SGIV-infected cells using a Coulter EPICS Elite ESP flow cytometer, whereas Cho and Kim [145] developed a protein chip based on surface plasmon resonance imaging (SPRI) to detect iridovirus antibodies using a recombinant 50 kDa fragment of the MCP protein as an antigen. In another study, Li et al. [146] developed a systematic evolution of ligands by exponential enrichment (SELEX) procedure for the in vitro selection of artificial ssDNA against SGIV, known as aptamers, that bind to targets through their stable three-dimensional structures. Electrophoretic mobility shift assays showed that aptamers bound SGIV specifically as evidenced by the lack of cross-reactivity with the softshell turtle iridovirus.

## 5. Epidemiology

### 5.1. Wide Host Range

As viral pathogens that can seriously endanger fish health, iridoviruses have an extremely wide host range. To date, reported fish hosts include various members of puffer, flounder and perciformes [31,33]. Nearly a hundred species of freshwater and marine fish species are susceptible to iridoviruses, which poses the risk of spreading infections from wild to farmed fish in aquaculture [6,33]. Studies carried out by Jeong et al. [147] showed the transmission of the *Pearl gourami* iridovirus (PGIV) from freshwater ornamental fish (*Pearl gourami*) to marine rock bream, suggesting that PGIV from freshwater ornamental fish may have crossed both the environmental and species barrier to infect marine fish such as rock bream. However, the prevalence of iridoviruses in wild reservoir hosts is unknown. The ability of wild reservoir hosts to transmit iridoviruses to farmed fish is also unknown. Furthermore, the factors that lead to iridoviruses crossing the species barrier from wild hosts to farmed fish are also unknown. In the absence of such information, it is difficult to develop intervention methods that can be used to prevent iridovirus infections passing from wild to farmed fish.

### 5.2. Persistent Carriers/Reservoirs

Persistent carriers serve as a source of recurrent outbreaks. For example, Hanson et al. [148] showed that LMBV persisted in a yellow waxy substance consisting of erythrocytes and eosinophils in the swim bladder of largemouth bass, serving as a source of infection when the fish became stressed, leading to recurrence of the disease. Choi et al. [149] detected RSIV in the heart, stomach, intestines, muscles, eyes and gills of asymptomatic rock bream while, strikingly, viral presence in the spleen was very low. Equally, Whittington and Reddacliff [150] found persistence of EHNV from clinically unaffected rainbow trout 63 days post exposure, whereas Kurobe et al. [151] reported the persistence of the Missouri River sturgeon iridovirus (MRSIV) among healthy pallid sturgeon (*Scaphirhynchus albus*) that recovered from clinical episodes after 8.5 months. Altogether, these studies show that fish surviving iridovirus outbreaks serve as virus carriers, becoming a source of infection in subsequent outbreaks.

### 5.3. Season and Temperature

Season and temperature are among the major factors that influence the occurrence of outbreaks caused by iridovirus diseases [144,145]. Several studies have shown that iridovirus diseases mostly occur in summer when water temperatures are high, reaching about 25–34 °C [152,153]. In an experimental challenge using ESV in sheatfish, mortality increased tremendously when the temperature increased above 25 °C [154]. Equally, Watson et al. [155] showed that water temperature had a significant effect on the increase in white sturgeon iridovirus (WSIV) disease outbreaks in juvenile white sturgeon in which mortality was higher during high temperatures. Wolf [156] showed that when the water temperature was maintained at 25 °C, symptoms caused by iridovirus infections in *Centrarchidae* lasted 10 days but were less pronounced for several weeks when the temperature was maintained at 12 °C. Furthermore, Smith et al. [157] showed that outbreaks caused by LMBV in largemouth bass were most prevalent during seasons of high water temperatures, in line with Grant et al. [158], who showed that experimentally infected largemouth bass with LMBV had higher mortality at 30 °C than at 25 °C. Similarly, ISKNV in Chinese perch (*S. chuatsi*) caused high mortality at temperatures above 25 °C and ISKN only occurred at temperatures above 20 °C [159]. Altogether, these studies showed that an increase in mortality due to iridovirus infections is associated with seasons of high temperatures.

### 5.4. Stress and Stocking Density

Stress events can affect the timing and severity of outbreaks caused by iridoviruses [160] while high stocking density has been linked to a high transmission index of viral diseases in aquaculture [161,162]. Higher stocking density, greater fluctuation of water temperature and low water flow have been associated with higher mortality in white sturgeon exposed to WSIV [163]. Drennan [164] evaluated the impact of stocking density on juvenile white sturgeon (*Acipenser transmontanus*) and showed that fish reared at density >3 g/L exhibited increasing signs of disease and mortality after exposure to WSIV. Inendino et al. [165] also showed that largemouth bass reared at a high stocking density had higher mortality rates, elevated viral loads, and reduced body condition compared with fish held at low density after exposure to LMBV. They also observed that rapid fluctuations in the concentrations of dissolved substances such as ammonia, nitrite, and nitrate had a greater impact on sensitivity to viral infection. Altogether, these findings show that stress factors associated with poor quality water and high stocking density lead to high mortality in iridovirus infections.

### 5.5. Viral Transmission

Iridovirus virions are mostly transmitted horizontally in fish culture. Various challenge studies have been carried out using cohabitation challenge exposure demonstrating the horizontal transmission of iridoviruses [147,166]. For LCDV, horizontal transmission has been linked to virus entry through external surfaces like gills and skin fissures [156,167]. Horizontal transmission is also considered to be the most important method of ENV transmission although in most challenge experiments the virus is administered by injection in fish rather than cohabitation [142,168,169]. Transmission of WSIV and MRSIV by cohabitation under experiments conditions also demonstrate the universality of water-borne transmission of iridoviruses from subclinical infected fish to susceptible fish [151,170]. Observation of fish erythrocytes infected by ENV in isopods showed that vector transmission should be considered [143]. Although vertical transmission of iridoviruses has not been confirmed, Georgiadis et al. [160,171] hypothesized that vertical transmission of WSIV may also occur in wild-caught breeding fish in which eggs from infected broodstock could serve as a source of infection to the new progeny rendering eggs to be the most important risk factor associated with vertical transmission. Similarly, Hedrick and LaPatra et al. [172,173] hypothesized that WSIV was introduced into white sturgeon farms from wild broodstock by vertical transmission of infected eggs during the early days of sturgeon farming. However, there is need for more studies to consolidate these obsevrations.

## 6. Prevention and Control of Disease

### 6.1. Biosecurity Control Measures

Given the ability of iridoviruses to cause high mortality in farmed fish, there is a need for the implementation of effective biosecurity measures to prevent the occurrence of outbreaks on fish farms. Husbandry practices such as introducing disease-free adult fish or uncontaminated eggs at the start of each production cycle; the implementation of the all-in-all-out principle; avoiding stress-inducing factors such as the use of poor quality water, avoiding overcrowding and the use of high-stocking densities; the implementation of hygiene measures on fish farms; the timely removal of moribund and dead fish from stock; the use of protective clothing when working on fish as well as disinfection of utensils and other fish-handling equipment can help reduce transmission of iridoviruses on fish farms. It is vital to acquire eggs and fry from disease-free broodstock and to ensure that broodstock are screened regularly for the absence of iridovirus infections. The timely diagnosis of disease outbreaks followed by notification of the relevant authorities is important. Where outbreaks have been reported, it is vital to leave the culture tanks fallow or other fish culture facilities, followed by their disinfection and physical inactivation by heating, fumigation or use of other inactivation methods. Biosecurity measures used for other viral diseases in aquaculture can also be used as previously described [161,162].

### 6.2. Physical and Chemical Properties of Iridoviruses

A good understanding of the physical and chemical properties of iridoviruses infecting finfish can serve as a guide in selecting effective disinfectants and the physical conditions needed for virus denaturation when applying biosecurity measures. For example, the optimal temperature for LMBV replication has been shown to be 30 °C, so high temperatures > 60 °C can be used for virus denaturation [76]. Piaskoski et al. [76] showed that LMBV was sensitive to ether treatment, which reduced its infectivity in fish, but was stable at pH 3–9 for 12 h at 4 °C. Lowering of the water temperature by 2–5 °C significantly reduced mortality from 7 to 3% in largemouth bass exposed to LMBV [174]. He et al. [159] and Fusianto et al. [175] showed that iodine was not effective in inactivating ISKNV at the different concentrations tested while potassium permanganate inactivated ISKNV at >100 ppm, formalin at 2000 ppm, sodium hypochlorite at 200 ppm, quaternary ammonium at 650 ppm and Virkon at 1%. He et al. [159] also showed that pH 3.0 and pH 7.0 were not effective for ISKNV inactivation but pH 11 inactivated the virus after 30 min. Temperatures > 50 °C inactivated ISKNV but it was not inactivated at temperatures < 40 °C. As for RSIV, it was shown to be sensitive to ether, chloroform and formalin treatment [176]. Thus, the chemical and physical properties for other iridoviruses can be determined for use in the disinfection and denaturation of the viruses as a control measure to prevent transmission among host species.

### 6.3. Vaccination

Vaccination is considered the most effective disease-control strategy, capable of preventing the occurrence of outbreaks and the spread of iridoviruses. As shown in Table 3, different experimental vaccines have been developed and tested for megalocytiviruses, ranaviruses and lymphocystiviruses. In general, these vaccines can be divided into two categories, namely the replicative and non-replicative vaccines. As pointed out previously [177], non-replicative vaccines are considered safe because they do not pose the risk of reverting to virulence. They produce antibodies against virus found in extracellular compartments such as the circulatory system. They only evoke humoral immune responses that do not last for a long duration, given that their antigens are not replicative. Hence, they require adjuvants to prolong their slow release from injection sites in order to generate a long duration of immune response. Non-replicative iridovirus vaccines tested under experimental conditions mostly consist of inactivated whole virus and subunit vaccines. As shown in Table 3, experimental DNA vaccines, inactivated and recombinant vaccines have been developed for ISKNV, RSIV, TRBIV, OSGIV and RBIV among the *Megalocytiviruses*, and for LMBV, SGIV and TGIV among the *Ranaviruses*. Table 3 shows experimental vaccines developed for *Lymphocystiviruses* mainly consisting of DNA vaccines. Different vectors have been used for the production of recombinant vaccines using the MCP as the protective antigen for all three genera, namely *Megalocytiviruses, Ranaviruses and Lymphocystiviruses*. In spite of this, only a few commercial vaccines are currently in use and these include the RSIV formalin-inactivated vaccine produced by the Research Foundation for Microbial disease in Japan [178,179] and the AQUAVAC IridoV produced by Merck Animal Health (USA) licensed for use in Singapore [179].

As for replicative vaccines, they have the advantage of evoking both the cell-mediated immune response able to eliminate virus-infected cells and humoral immune responses able to neutralize virus found in extracellular compartments such as the circulatory system. Although attenuation of iridoviruses to produce live vaccines has not been documented, the lowering of temperature as a method of reducing viral virulence to evoke cell-mediated and humoral responses was reported [199,200]. However, the most explored method for producing replicative iridovirus vaccines tested under experimental conditions is the production of DNA vaccines using the MCP protein expressed in recombinant vectors (Table 3). Thus, experimental DNA vaccines have been developed and tested for different iridoviruses although there is no documented commercial DNA vaccine currently in use.

## 7. Conclusions

Fish iridovirus diseases are a worldwide problem adversely affecting the expansion of global aquaculture. The three genera causing diseases in fish consist of *Ranaviruses*, *Megalocytiviruses* and *Lymphocystiviruses*. Thus, these genera have attracted a lot of research interest in recent years leading to whole genome sequencing of the major iridovirus species causing disease in aquaculture. Although considerable progress has been made in developing cell-lines for virus isolation, coupled with ongoing advances in developing molecular tools for timely disease diagnosis, the regulatory mechanism of virus replication and transcription for most iridoviruses is still unknown. Thus, the functional roles of different genes encoded in the iridovirus genomes are still unknown. As such, the pathogenicity mechanisms leading to disease establishment have not been elucidated. Although the MCP is the protein most widely used for developing diagnostic tools and recombinant vaccines, the immunogenic properties of other proteins are unknown. In spite of this, there has been considerable progress made in vaccine research, which has led to licensure of some of the vaccines. However, given their ability to infect a wide range of fish species coupled with their ability to cause high mortality in infected fish, there is still urgent need for the development of more protective vaccines against iridoviruses in order to reduce their adverse effects in aquaculture.

## Figures and Tables

**Table 1:** Comparison of genome organization of lymphocystiviruses, megalocytiviruses and ranaviruses.

Genus	Viral Pathogen	Abbrev	Size (bp)	No ORF	ORF Size (aa)	G + C% Content	GenBank Acc No.
*Lymphocystivirus*	Lymphocystis disease virus-1	LCDV-1	102,653	195	40~1199	29	L63545
	Lymphocystis disease virus-C	LCDV-C	186,250	240	40~1193	27	AY380826
*Megalocytivirus*	Infectious spleen and kidney necrosis virus	ISKNV	111,362	125	40~1208	55	AF371960
	Rock bream iridovirus	RBIV	112,080	100	50~1253	53	AY532606
	Red sea bream iridovirus	RSIV	112,414	114	40~1168	53	MT798582
	Orange spotted grouper iridovirus	OSGIV	112,636	121	40~1168	54	AY894343
	Turbot reddish body iridovirus	TRBIV	110,104	115	40~1168	55	GQ273492
	Large yellow croaker iridovirus	LYCIV	111,760	126	ND	ND	AY779031
*Ranavirus*	Enzootic hematopoietic necrosis virus	EHNV	127,011	100	ND	54	FJ433873
	*Rana grylio* iridovirus	RGV	105,791	106	ND	55	JQ654586
	European sheatfish virus	ESV	127,732	133	ND	54	JQ724856
	Singapore grouper iridovirus	SGIV	140,131	162	40~1268	49	AY521625
	Grouper iridovirus	GIV	139,793	120	60~1268	49	AY666015

**Table 2:** Cell lines used for the culture of lymphocystiviruses, megalocytiviruses and ranaviruses.

Viral Pathogen	Abbrev	Cell Line Name	Abbrev	Reference
Largemouth bass virus	LMBV	Fathead minnow	FHM	[76]
		Bluegill fry *Lepomis macrochirus*	BF-2	[77]
		Epithelioma papulosum cyprini	EPC	[78]
		Channel catfish ovary	CCO	[76]
		Chinook Salmon embryo	CHSE-214	[76]
		Largemouth bass fin (*Micropterus salmoides*)	MsF	[79]
		Largemouth bass heart (*Micropterus salmoides*)	MsH	[80]
Enzootic hematopoietic necrosis virus	EHNV	Fathead minnow	FHM	[81]
		Bluegill fry *Lepomis macrochirus*	BF-2	[77]
		Chinook Salmon embryo	CHSE-214	[81]
European catfish virus	ECV	Fathead minnow	FHM	[82]
		Bluegill fry *Lepomis macrochirus*	BF-2	[77]
		Epithelium papulosum cyprinid	EPC	[82]
		Channel catfish ovary	CCO	[23]
Singapore grouper iridovirus	SGIV	*Epinephelus akaara* grouper kidney	EAGK	[83]
		*Epinephelus akaara* grouper spleen	EAGS	[83]
		*Epinephelus akaara* grouper swim bladder	EAGSB	[83]
		Grouper embryonic cells	GEC	[84]
		Grouper head kidney cell	ELHK	[85]
Grouper iridovirus	GIV	Barramundi muscle	BM	[86]
		Barramundi swim bladder	BSB	[84]
		Grouper eye, heart and swim bladder	GE	[84]
		Grouper fin	GF	[84]
		Grouper heart	GH	[84]
		Grouper swim bladder	GSB	[84]
		Orange-spotted grouper spleen	GS-1	[87]
		Grouper *Epinephelus awoara* kidney	GK	[88]
		Grouper *Epinephelus awoara* liver	GL	[88]
Infectious spleen and kidney virus	ISKNV	Mandarin fish fry	MFF-1	[89]
		Chinese perch brain cell line	CPB	[90]
		Chinese perch brain cells	CPB	[91]
		Epithelioma papulosum cyprini	EPC	[45]
		Fathead minnow	FHM	[45]
		Epithelioma papulosum cyprini	EPC	[45]
		Bluegill fry *Lepomis macrochirus*	BF-2	[45]
		Orange-spotted grouper spleen	GS-1	[87]
Red sea bream iridovirus	RSIV	Grunt fin cells	GF	[92]
		Red spotted grouper embryo	KRE-3	[93]
		Bluegill fry *Lepomis macrochirus*	BF-2	[77]
		Hirame natural embryo cells	HINAE	[94]
		Spotted knifejaw (*Oplegnathus punctatus*)	SKF-9	[95]
		Red sea bream fin tail	CRF-1	[36]
		Rock bream *Oplegnathus fasciatus* embryo	RoBE-4	[96]
		Splenic cell line from sea bass *Lates calcarifer*	SISS	[97]
Rock bream iridovirus	RBIV	Grunt fin cells	GF	[98]
		Bluegill fry *Lepomis macrochirus*	BF-2	[99]
Turbot reddish body iridovirus	TRBIV	Turbot (*Scophthalmus maximus)* fin cell line	TF	[100]
		Epithelioma papulosum cyprini	EPC	[45]
		Fathead minnow	FHM	[45]
		Bluegill fry *Lepomis macrochirus*	BF-2	[45]
		Turbot (*Scophthalmus maximus*) kidney cells	TK	[101,102]
		Brown-marbled grouper fin cell line	bmGF-1	[103]
		(*Cynoglossus semilaevis*) gonad cell	CSGC	[104]
Orange spotted grouper iridovirus	OSGIV	Mandarin fish fry	MFF-1	[105]
		*L. crocea* embryo	YCE1	[106]
		Bluegill fry *Lepomis macrochirus*	BF-2	[107]
Lymphocystis disease virus	LCDV-C	*Sparus aurata* fibroblast	SAF-1	[108]
		Epithelioma papulosum cyprini	EPC	[109]
		Bluegill fry *Lepomis macrochirus*	BF-2	[109]
		Chinook Salmon embryo	CHSE-214	[109]
		Turbot (*Scophthalmus maximus*) kidney	TK	[101,102]
		Brown-marbled grouper fin cell line	bmGF-1	[103]
		*Cynoglossus semilaevis* gonad cell	CSGC	[104]

**Table 3:** Experimental vaccines developed against megalocytiviruses, ranaviruses and lymphocystiviruses.

Pathogen	Host Species	Vaccine Types	Reference
ISKNV	Mandarin fish (*Siniperca chuatsi)*	Inactivated vaccine	[180]
		SWCNTs subunit vaccine (SWCNTs-M-MCP)	[181]
		DNA plasmid containing *mcp*	[91]
		Single-walled carbon nanotubes DNA ORF093-	[182]
		Formalin-killed cell vaccine	[183]
		Early protein ORF086	[184]
		Mannose-modified subunit vaccine	[185]
RSIV	Red seabream (*Pagrus major*)	MCP-DNA vaccine	[186]
		Formalin-inactivated RSIV vaccine	[186]
		Formalin-killed viral vaccine	[178]
		Yeast *Saccharomyces cerevisiae* subunit vaccine	[187]
TRBIV	Turbot (*Scophthalmus maximus* L.)	Formalin and aluminum hydroxide inactivated	[188]
		Chitosan nanoparticle plasmids encoding DNA (pDNA-CS-NPs)	[189]
		Major capsid protein (MCP) DNA vaccine	[190]
Orange-spotted grouper iridovirus (OSGIV)	Giant grouper *(Epinephelus lanceolatus)*	Subunit oral and microencapsulation vaccine	[191]
RBIV	Japanese flounder (*Paralichthys olivaceus*) and turbot (*Scophthalmus maximus*)	DNA vaccine encoding myristoylated membrane protein (MMP)	[192]
		DNA vaccine with MCP capsid	[193]
LMBV	Largemouth bass (*Micropterus salmoides*)	DNA vaccine	[78]
		recombinant baculovirus vector vaccine (BacMCP)	[194]
SGIV	Orange-spotted grouper (*Epinephelus coioides*)	*β*-propiolactone (BPL) inactivated virus	[195]
		Formalin inactivated virus	[195]
		DNA vaccines	[196]
		SGIV *ORF19R* (*SGIV-19R*) viral membrane protein	[197]
Grouper iridovirus of Taiwan (TGIV)	Grouper (*Epinephelus coioides*)	Recombinant MCP Vaccine	[195]
LCDV	Japanese flounder (*Paralichthys olivaceus*)	DNA vaccine	[196]
		Oral poly (DL-lactide-co-glycolide) microcapsules	[197]
		Alginate microspheres DNA vaccine	[198]

## Data Availability

Not applicable.

## References

[1] Whittington R.J., Becker J.A., Dennis M.M. (2010). Iridovirus infections in finfish—Critical review with emphasis on ranaviruses. J. Fish Dis..

[2] Chen X., Qiu L., Wang H., Zou P., Dong X., Li F., Huang J. (2019). Susceptibility of Exopalaemon carinicauda to the infection with shrimp hemocyte iridescent virus (SHIV 20141215), a strain of decapod iridescent virus 1 (DIV1). Viruses.

[3] Chinchar V.G., Hyatt A., Miyazaki T., Williams T. (2009). Family Iridoviridae: Poor viral relations no longer. Curr. Top. Microbiol. Immunol..

[4] Chinchar V., Yang F., Huang J., Williams T., Whittington R., Jancovich J., Subramaniam K., Waltzek T., Hick P., Ince I. One new genus with one new species in the subfamily Betairidovirinae. ICTV Taxonomic Proposal to the Iridoviridae Study Group of International Committee for Taxonomy of Viruses, 2018.004D ICTV 2018. https://ictv.global/filebrowser/download/4955.

[5] Chinchar V.G., Yu K.H., Jancovich J.K. (2011). The Molecular Biology of Frog Virus 3 and other Iridoviruses Infecting Cold-Blooded Vertebrates. Viruses.

[6] Kurita J., Nakajima K. (2012). Megalocytiviruses. Viruses.

[7] Graham D., Curran W., Rowley H., Cox D., Cockerill D., Campbell S., Todd D. (2002). Observation of virus particles in the spleen, kidney, gills and erythrocytes of Atlantic salmon, *Salmo salar* L., during a disease outbreak with high mortality. J. Fish Dis..

[8] Grizzle J.M., Altinok I., Fraser W.A., Francis-Floyd R. (2002). First isolation of largemouth bass virus. Dis. Aquat. Org..

[9] Plumb J.A., Grizzle J.M., Young H.E., Noyes A.D., Lamprecht S. (1996). An Iridovirus Isolated from Wild Largemouth Bass. J. Aquat. Anim. Health.

[10] Goldberg T. (2002). Largemouth bass virus: An emerging problem for warmwater fisheries?. Am. Fish. Soc. Symp..

[11] Chinchar V.G. (2002). Ranaviruses (family Iridoviridae): Emerging cold-blooded killers. Arch. Virol..

[12] Ohlemeyer S., Holopainen R., Tapiovaara H., Bergmann S.M., Schütze H. (2011). Major capsid protein gene sequence analysis of the Santee-Cooper ranaviruses DFV, GV6, and LMBV. Dis. Aquat. Org..

[13] Plumb J.A., Zilberg D. (1999). The lethal dose of largemouth bass virus in juvenile largemouth bass and the comparative susceptibility of striped bass. J. Aquat. Anim. Health.

[14] Zilberg D., Grizzle J.M., Plumb J.A. (2000). Preliminary description of lesions in juvenile largemouth bass injected with largemouth bass virus. Dis. Aquat. Org..

[15] Langdon J.S., Humphrey J.D., Williams L.M., Hyatt A.D., Westbury H.A. (1986). First virus isolation from Australian fish: An iridovirus-like pathogen from redfin perch, *Perca fluviatilis* L. J. Fish Dis..

[16] Hyatt A.D., Eaton B.T., Hengstberger S., Russel G. (1991). Epizootic haematopoietic necrosis virus: Detection by ELISA, immunohistochemistry and immunoelectron-microscopy. J. Fish Dis..

[17] Hedrick R.P., Mcdowell T.S., Ahne W., Torhy C., Kinkelin P.D. (1992). Properties of three iridovirus-like agents associated with systemic infections of fish. Dis. Aquat. Org..

[18] Hengstberger S.G., Hyatt A.D., Speare R.S., Coupar B. (1993). Comparison of epizootic haematopoietic necrosis and Bohle iridoviruses, recently isolated Australian iridoviruses. Dis. Aquat. Org..

[19] Hyatt A.D., Gould A.R., Zupanovic Z., Cunningham A.A., Hengstberger S., Whittington R.J., Kattenbelt J., Coupar B.E.H. (2000). Comparative studies of piscine and amphibian iridoviruses. Arch. Virol..

[20] Becker J.A., Gilligan D., Asmus M., Tweedie A., Whittington R.J. (2019). Geographic Distribution of Epizootic haematopoietic necrosis virus (EHNV) in Freshwater Fish in South Eastern Australia: Lost Opportunity for a Notifiable Pathogen to Expand Its Geographic Range. Viruses.

[21] Langdon J.S., Humphrey J.D. (1987). Epizootic haematopoietic necrosis, a new viral disease in redfin perch, *Perca fluviatilis* L., in Australia. J. Fish Dis..

[22] Whittington R., Becker J., Tweedie A., Gilligan D., Asmus M. (2010). Susceptibility of previously untested basin fish species to EHN virus and epidemiology of EHN virus in the wild. Native Fish Forum 2010.

[23] Ahne W., Schlotfeldt H., Thomsen I. (1989). Fish viruses: Isolation of an icosahedral cytoplasmic deoxyribovirus from sheatfish (*Silurus glanis*). J. Vet. Med. Ser. B.

[24] Marsh I.B., Whittington R.J., O’Rourke B., Hyatt A.D., Chisholm O. (2002). Rapid differentiation of Australian, European and American ranaviruses based on variation in major capsid protein gene sequence. Mol. Cell. Probes.

[25] Chua F.H., Ng M.L., Ng K.L., Loo J.J., Wee J.Y. (1994). Investigation of outbreaks of a novel disease, ‘Sleepy Grouper Disease’, affecting the brown-spotted grouper, *Epinephelus tauvina* Forskal. J. Fish Dis..

[26] Chang S.F., Ngoh-Lim G.H., Kueh L.F.S., Qin Q.W., Seng E.K., Sin Y.M. (2002). Initial investigations into two viruses isolated from marine food fish in Singapore. Vet. Rec..

[27] Qin Q.W., Lam T.J., Sin Y.M., Shen H., Chang S.F., Ngoh G.H., Chen C.L. (2001). Electron microscopic observations of a marine fish iridovirus isolated from brown-spotted grouper, *Epinephelus tauvina*. J. Virol. Methods.

[28] Qin Q., Chang S., Ngoh-Lim G., Gibson-Kueh S., Shi C., Lam T. (2003). Characterization of a novel ranavirus isolated from grouper *Epinephelus tauvina*. Dis. Aquat. Org..

[29] Chen L.M., Wang F., Song W., Hew C.L. (2006). Temporal and differential gene expression of Singapore grouper iridovirus. J. Gen. Virol..

[30] Tsai C.T., Ting J.W., Wu M.H., Wu M.F., Guo I.C., Chang C.Y. (2005). Complete Genome Sequence of the Grouper Iridovirus and Comparison of Genomic Organization with Those of Other Iridoviruses. J. Virol..

[31] Kawakami H., Nakajima K. (2002). Cultured fish species affected by red sea bream iridoviral disease from 1996 to 2000. Fish Pathol..

[32] Matsuoka S., Inouye K., Nakajima K. (1996). Cultured fish species affected by red sea bream iridoviral disease from 1991 to 1995. Fish Pathol..

[33] MacLachlan N.J., Dubovi E.J. (2017). Chapter 2—Virus replication. Fenner’s Veterinary Virology.

[34] Kwon W.J., Choi J.C., Hong S., Kim Y.C., Jeong M.G., Min J.G., Jeong J.B., Kim K.I., Do Jeong H. (2020). Development of a high-dose vaccine formulation for prevention of megalocytivirus infection in rock bream (*Oplegnathus fasciatus*). Vaccine.

[35] Inouye K., Yamano K., Maeno Y., Nakajima K., Matsuoka M., Wada Y., Sorimachi M. (1992). Iridovirus Infection of Cultured Red Sea Bream, *Pagrus major*. Fish Pathol..

[36] Imajoh M., Ikawa T., Oshima S.I. (2007). Characterization of a new fibroblast cell line from a tail fin of red sea bream, *Pagrus major*, and phylogenetic relationships of a recent RSIV isolate in Japan. Virus Res..

[37] Jung S.J., Oh M.J. (2000). Iridovirus-like infection associated with high mortalities of striped beakperch, *Oplegnathus fasciatus* (Temminck et Schlegel), in southern coastal areas of the Korean peninsula. J. Fish Dis..

[38] Zhang M., Xiao Z.h., Hu Y.u., Sun L. (2012). Characterization of a megalocytivirus from cultured rock bream, *Oplegnathus fasciatus* (Temminck & Schlege), in China. Aquac. Res..

[39] He J.G., Deng M., Weng S.P., Li Z., Chan S.M. (2002). Complete Genome Analysis of the Mandarin Fish Infectious Spleen and Kidney Necrosis Iridovirus. Virology.

[40] Rimmer A.E., Whittington R.J., Tweedie A., Becker J.A. (2017). Susceptibility of a number of Australian freshwater fishes to dwarf gourami iridovirus (Infectious spleen and kidney necrosis virus). J. Fish Dis..

[41] Wang Q., Zeng W.W., Li K.B., Chang O.Q., Liu C., Wu G.H., Shi C.B., Wu S.Q. (2011). Outbreaks of an iridovirus in marbled sleepy goby, *Oxyeleotris marmoratus* (Bleeker), cultured in southern China. J. Fish Dis..

[42] Weber E.S., Waltzek T.B., Young D.A., Twitchell E.L., Gates A.E., Vagelli A., Risatti G.R., Hedrick R.P., Frasca S. (2009). Systemic iridovirus infection in the Banggai cardinalfish (*Pterapogon kauderni* Koumans 1933). J. Vet. Diagn. Investig..

[43] Shi C.Y., Wang Y.G., Yang S.L., Huang J., Wang Q.Y. (2004). The first report of an iridovirus-like agent infection in farmed turbot, *Scophthalmus maximus*, in China. Aquaculture.

[44] Do J., Cha S., Kim J., An E., Lee N., Choi H., Lee C., Park M., Kim J., Kim Y. (2005). Phylogenetic analysis of the major capsid protein gene of iridovirus isolates from cultured flounders *Paralichthys olivaceus* in Korea. Dis. Aquat. Org..

[45] Go J., Waltzek T.B., Subramaniam K., Yun S.C., Groff J.M., Anderson I.G., Chong R., Shirley I., Schuh J.C.L., Handlinger J.H. (2016). Detection of infectious spleen and kidney necrosis virus (ISKNV) and turbot reddish body iridovirus (TRBIV) from archival ornamental fish samples. Dis. Aquat. Org..

[46] Koda S.A., Subramaniam K., Francis-Floyd R., Yanong R.P., Frasca S., Groff J.M., Popov V.L., Fraser W.A., Yan A., Mohan S. (2018). Phylogenomic characterization of two novel members of the genus *Megalocytivirus* from archived ornamental fish samples. Dis. Aquat. Org..

[47] Huang S.M., Tu C., Tseng C.H., Huang C.C., Chou C.C., Kuo H.C., Chang S.K. (2011). Genetic analysis of fish iridoviruses isolated in Taiwan during 2001–2009. Arch. Virol..

[48] Chen X.H., Lin K.B., Wang X.W. (2003). Outbreaks of an iridovirus disease in maricultured large yellow croaker, *Larimichthys crocea* (Richardson), in China. J. Fish Dis..

[49] Wang G., Luan Y., Wei J., Li Y., Shi H., Cheng H., Bai A., Xie J., Xu W., Qin P. (2022). Genetic and Pathogenic Characterization of a New Iridovirus Isolated from Cage-Cultured Large Yellow Croaker (*Larimichthys crocea*) in China. Viruses.

[50] Noga E.J. (2010). Fish Disease: Diagnosis and Treatment.

[51] Lowe J. (1874). Fauna and flora of Norfolk. Part IV. Fishes. Trans. Norfolk Norwich Nat. Soc..

[52] Walker R. (1962). Fine structure of lymphocystis virus of fish. Virology.

[53] Tidona C.A., Darai G. (1997). The Complete DNA Sequence of Lymphocystis Disease Virus. Virology.

[54] Williams T., Barbosa-Solomieu V., Chinchar V.G. (2005). A decade of advances in iridovirus research. Adv. Virus Res..

[55] Zhang Q.Y., Xiao F., Xie J., Li Z.Q., Gui J.F. (2004). Complete Genome Sequence of Lymphocystis Disease Virus Isolated from China. J. Virol..

[56] Kawato S., Nozaki R., Hirono I., Kondo H. (2021). Genome Sequence of Lymphocystis Disease Virus 2 LCDV-JP_Oita_2018, Isolated from a Diseased Japanese Flounder (*Paralichthys olivaceus*) in Japan. Microbiol. Resour. Announc..

[57] Doszpoly A., Kaján G.L., Puentes R., Perretta A. (2020). Complete genome sequence and analysis of a novel lymphocystivirus detected in whitemouth croaker (*Micropogonias furnieri*): Lymphocystis disease virus 4. Arch. Virol..

[58] Heppell J., Berthiaume L. (1992). Ultrastructure of lymphocystis disease virus (LDV) as compared to frog virus 3 (FV 3) and chilo iridescent virus (CIV): Effects of enzymatic digestions and detergent degradations. Arch. Virol..

[59] Nicholson B.L., Reno P.W. (1981). Viral erythrocytic necrosis (VEN) in marine fishes. Fish Pathol..

[60] Robin J., Laperrière A., Berthiaume L. (1986). Identification of the glycoproteins of lymphocystis disease virus (LDV) of fish. Arch. Virol..

[61] Grayfer L., Andino F.D.J., Chen G., Chinchar G.V., Robert J. (2012). Immune Evasion Strategies of Ranaviruses and Innate Immune Responses to These Emerging Pathogens. Viruses.

[62] Eaton H.E., Ring B.A., Brunetti C.R. (2010). The Genomic Diversity and Phylogenetic Relationship in the Family Iridoviridae. Viruses.

[63] Delius H., Darai G., Flügel R.M. (1984). DNA Analysis of Insect Iridescent Virus 6: Evidence for Circular Permutation and Terminal Redundancy. J. Virol..

[64] Mahy B.W., Van Regenmortel M.H. (2008). Encyclopedia of Virology.

[65] Hossain M., Song J.Y., Kitamura S.I., Jung S.J., Oh M.J. (2008). Phylogenetic analysis of lymphocystis disease virus from tropical ornamental fish species based on a major capsid protein gene. J. Fish Dis..

[66] Eaton H.E., Metcalf J., Penny E., Tcherepanov V., Upton C., Brunetti C.R. (2007). Comparative genomic analysis of the familyIridoviridae: Re-annotating and defining the core set of iridovirus genes. Virol. J..

[67] de Matos A.P., Caeiro M.F., Papp T., Matos B.A., Correia A.C., Marschang R.E. (2011). New viruses from *Lacerta monticola* (Serra da Estrela, Portugal): Further evidence for a new group of nucleo-cytoplasmic large deoxyriboviruses. Microsc. Microanal..

[68] Huang Y., Huang X., Cai J., Ye F., Guan L., Liu H., Qin Q. (2011). Construction of green fluorescent protein-tagged recombinant iridovirus to assess viral replication. Virus Res..

[69] Tidona C.A., Schnitzler P., Kehm R., Darai G. (1998). Is the Major Capsid Protein of Iridoviruses a Suitable Target for the Study of Viral Evolution?. Virus Genes.

[70] Kim W.S., Oh M.J., Jung S.J., Kim Y.J., Kitamura S.I. (2005). Characterization of an iridovirus detected from cultured turbot *Scophthalmus maximus* in Korea. Dis. Aquat. Org..

[71] Hershberger P., Hart A., Gregg J., Elder N., Winton J. (2006). Dynamics of viral hemorrhagic septicemia, viral erythrocytic necrosis and ichthyophoniasis in confined juvenile Pacific herring *Clupea pallasii*. Dis. Aquat. Org..

[72] Paperna I., de Matos A.A. (1993). Erythrocytic viral infections of lizards and frogs: New hosts, geographical locations and description of the infection process. Ann. Parasitol. Hum. Comp..

[73] St M.J., Young J., Williams L.M. (2005). Epizootic haematopoietic necrosis virus (EHNV): Growth in fish cell lines at different temperatures. Bull.-Eur. Assoc. Fish Pathol..

[74] Mcclenahan S.D., Beck B.H., Grizzle J.M. (2005). Evaluation of Cell Culture Methods for Detection of Largemouth Bass Virus. J. Aquat. Anim. Health.

[75] Gong J., Huang Y., Huang X., Ouyang Z., Guo M., Qin Q. (2011). Establishment and characterization of a new cell line derived from kidney of grouper, *Epinephelus akaara* (Temminck & Schlegel), susceptible to Singapore grouper iridovirus (SGIV). J. Fish Dis..

[76] Piaskoski T.O., Plumb J.A., Roberts S.R. (1999). Characterization of the largemouth bass virus in cell culture. J. Aquat. Anim. Health.

[77] Hoffman G., Dunbar C., Wolf K., Zwillenberg L. (1969). Epitheliocystis, a new infectious disease of the bluegill (*Lepomis macrochirus*). Antonie van Leeuwenhoek.

[78] Yi W., Zhang X., Zeng K., Xie D., Song C., Tam K., Liu Z., Zhou T., Li W. (2020). Construction of a DNA vaccine and its protective effect on largemouth bass (*Micropterus salmoides*) challenged with largemouth bass virus (LMBV). Fish Shellfish Immunol..

[79] Yang J., Xu W., Wang W., Pan Z., Qin Q., Huang X., Huang Y. (2022). Largemouth Bass Virus Infection Induced Non-Apoptotic Cell Death in MsF Cells. Viruses.

[80] Zeng W., Dong H., Chen X., Bergmann S.M., Yang Y., Wei X., Tong G., Li H., Yu H., Chen Y. (2022). Establishment and characterization of a permanent heart cell line from largemouth bass *Micropterus salmoides* and its application to fish virology and immunology. Aquaculture.

[81] LaPatra S.E. (2014). 2.2. 4 Infectious hematopoietic necrosis. AFSFHS. Fish Health Section Blue Book: Suggested Procedures for the Detection and Identification of Certain Finfish and Shellfish Pathogens.

[82] Ariel E., Nicolajsen N., Christophersen M.-B., Holopainen R., Tapiovaara H., Jensen B.B. (2009). Propagation and isolation of ranaviruses in cell culture. Aquaculture.

[83] Huang X., Huang Y., Sun J., Han X., Qin Q. (2009). Characterization of two grouper *Epinephelus akaara* cell lines: Application to studies of Singapore grouper iridovirus (SGIV) propagation and virus–host interaction. Aquaculture.

[84] Lai Y.S., John J., Lin C.H., Guo I.C., Chen S.C., Fang K., Lin C.H., Chang C.Y. (2003). Establishment of cell lines from a tropical grouper, *Epinephelus awoara* (Temminck & Schlegel), and their susceptibility to grouper irido-and nodaviruses. J. Fish Dis..

[85] Liu Z., Zhang X., Zhang Y., Qin Q., Huang X., Huang Y. (2021). Establishment of a cell line from the head kidney of giant grouper (*Epinephelus lanceolatus*) and its susceptibility to fish viruses. Aquac. Rep..

[86] Lai Y.S., Chiou P., Chen W.J., Chen Y.C., Chen C.W., Chiu I.S., Chen S.D., Cheng Y.H., Chang C.Y. (2008). Characterization of apoptosis induced by grouper iridovirus in two newly established cell lines from barramundi, *Lates calcarifer* (Bloch). J. Fish Dis..

[87] Huang S.-M., Kuo S.-T., Kuo H.-C., Chang S.-K. (2018). Assessment of fish iridoviruses using a novel cell line GS-1, derived from the spleen of orange-spotted grouper *Epinephelus coioides* (Hamilton) and susceptible to ranavirus and megalocytivirus. J. Vet. Med. Sci..

[88] Lai Y.S., Murali S., Ju H.Y., Wu M.F., Guo I.C., Chen S.C., Fang K., Chang C.Y. (2000). Two iridovirus-susceptible cell lines established from kidney and liver of grouper, *Epinephelus awoara* (Temminck & Schlegel), and partial characterization of grouper iridovirus. J. Fish Dis..

[89] Dong C., Weng S., Shi X., Xu X., Shi N., He J. (2008). Development of a mandarin fish *Siniperca chuatsi* fry cell line suitable for the study of infectious spleen and kidney necrosis virus (ISKNV). Virus Res..

[90] Fu X., Li N., Lai Y., Luo X., Wang Y., Shi C., Huang Z., Wu S., Su J. (2015). A novel fish cell line derived from the brain of Chinese perch *Siniperca chuatsi*: Development and characterization. J. Fish Biol..

[91] Fu X., Li N., Lin Q., Guo H., Zhang D., Liu L., Wu S. (2014). Protective immunity against infectious spleen and kidney necrosis virus induced by immunization with DNA plasmid containing mcp gene in Chinese perch *Siniperca chuatsi*. Fish Shellfish Immunol..

[92] Clem L.W., Moewus L., Michael Sigel M. (1961). Studies with cells from marine fish in tissue culture. Proc. Soc. Exp. Biol. Med..

[93] Nakajima K., Sorimachi M. (1994). Biological and physico-chemical properties of the iridovirus isolated from cultured red sea bream, *Pagrus major*. Fish Pathol..

[94] Kasai H., Yoshimizu M. (2001). Establishment of two Japanese flounder [*Paralichthys olivaceus*] embryo cell lines. Bull. Fish. Sci. Hokkaido Univ..

[95] Kawato Y., Yamashita H., Yuasa K., Miwa S., Nakajima K. (2017). Development of a highly permissive cell line from spotted knifejaw (*Oplegnathus punctatus*) for red sea bream iridovirus. Aquaculture.

[96] Oh S.-Y., Nishizawa T. (2016). Establishment of rock bream *Oplegnathus fasciatus* embryo (RoBE-4) cells with cytolytic infection of red seabream iridovirus (RSIV). J. Virol. Methods.

[97] Parameswaran V., Shukla R., Bhonde R., Hameed A.S. (2006). Splenic cell line from sea bass, *Lates calcarifer*: Establishment and characterization. Aquaculture.

[98] Zenke K., Kim K.H. (2008). Functional characterization of the RNase III gene of rock bream iridovirus. Arch. Virol..

[99] Kim Y.I., Ha Y.M., Nam Y.K., Kim K.H., Kim S.K. (2008). Production of polyclonal antibody against recombinant ORF 112 L of rock bream (*Oplegnathus fasciatus*) iridovirus (RBIV) and in vitro neutralization. J. Environ. Biol..

[100] Fan T.-J., Ren B.-X., Geng X.-F., Yu Q.-T., Wang L.-Y. (2010). Establishment of a turbot fin cell line and its susceptibility to turbot reddish body iridovirus. Cytotechnology.

[101] Wei Y.B., Fan T.J., Jiang G.J., Xu X.H., Sun A. (2010). A novel heart-cell line from brown-marbled grouper *Epinephelus fuscoguttatus* and its susceptibility to iridovirus. J. Fish Biol..

[102] Wang N., Wang X., Sha Z., Tian Y., Chen S. (2010). Development and characterization of a new marine fish cell line from turbot (*Scophthalmus maximus*). Fish Physiol. Biochem..

[103] Wei Y., Fan T., Jiang G., Sun A., Xu X., Wang J. (2009). Establishment of a novel fin cell line from Brown-marbled grouper, *Epinephelus fuscoguttatus* (Forsskål), and evaluation of its viral susceptibility. Aquac. Res..

[104] Zhang B., Wang X., Sha Z., Yang C., Liu S., Wang N., Chen S.-L. (2011). Establishment and characterization of a testicular cell line from the half-smooth tongue sole, *Cynoglossus semilaevis*. Int. J. Biol. Sci..

[105] Ma H., Peng C., Su Y., Feng J., Guo Z. (2016). Isolation of a Ranavirus-type grouper iridovirus in mainland China and comparison of its pathogenicity with that of a Megalocytivirus-type grouper iridovirus. Aquaculture.

[106] Zheng Z., Chi H., Liu X., Yang X., Chen X., Pan Y., Gong H. (2023). A new embryonic cell line YCE1 from large yellow croaker (*Larimichthys crocea*) and its susceptibility to large yellow croaker iridovirus. Aquaculture.

[107] Ao J., Chen X. (2006). Identification and characterization of a novel gene encoding an RGD-containing protein in large yellow croaker iridovirus. Virology.

[108] Valverde E.J., Borrego J.J., Castro D. (2016). Evaluation of an integrated cell culture RT-PCR assay to detect and quantify infectious lymphocystis disease virus. J. Virol. Methods.

[109] Alonso M., Ferro P., Garcia-Rosado E., Cano I., Lang T., Bergmann S., Borrego J. (2007). Comparison of lymphocystis disease virus (LCDV) isolates obtained from different marine fish species and geographical areas. Bull.-Eur. Assoc. Fish Pathol..

[110] Chinchar V.G., Mao J. (2000). Molecular diagnosis of iridovirus infections in cold-blooded animals. Semin. Avian Exot. Pet Med..

[111] Galli L., Pereira A., Márquez A., Mazzoni R. (2006). Ranavirus detection by PCR in cultured tadpoles (*Rana catesbeiana* Shaw, 1802) from South America. Aquaculture.

[112] Allender M.C., Abd-Eldaim M., Schumacher J., Mcruer D., Kennedy M. (2011). PCR Prevalence of Ranavirus in Free-Ranging Eastern Box Turtles (*Terrapene carolina carolina*) at Rehabilitation Centers in Three Southeastern US States. J. Wildl. Dis..

[113] Allender M.C., Bunick D., Mitchell M.A. (2013). Development and validation of TaqMan quantitative PCR for detection of frog virus 3-like virus in eastern box turtles (*Terrapene carolina carolina*). J. Virol. Methods.

[114] Gias E., Johnston C., Keeling S., Spence R.P., Mcdonald W.L. (2011). Development of real-time PCR assays for detection of megalocytiviruses in imported ornamental fish. J. Fish Dis..

[115] Jun K., Kazuhiro N., Ikuo H., Takashi A. (1998). Polymerase chain reaction (PCR) amplification of DNA of red sea bream iridovirus (RSIV). Fish Pathol..

[116] Jeong J.B., Park K.H., Kim H.Y., Hong S.H., Kim K.H., Chung J.K., Komisar J.L., Jeong H.D. (2004). Multiplex PCR for the diagnosis of red sea bream iridoviruses isolated in Korea. Aquaculture.

[117] Getchell R.G., Groocock G.H., Schumacher V.L., Grimmett S.G., Wooster G.A., Bowser P.R. (2007). Quantitative polymerase chain reaction assay for largemouth bass virus. J. Aquat. Anim. Health.

[118] Cano I., Ferro P., Alonso M.C., Bergmann S.M., R?Mer-Oberd?Rfer A., Garcia-Rosado E., Castro D., Borrego J.J. (2010). Development of molecular techniques for detection of lymphocystis disease virus in different marine fish species. J. Appl. Microbiol..

[119] Kitamura S.I., Jung S.J., Oh M.J. (2006). Differentiation of lymphocystis disease virus genotype by multiplex PCR. J. Microbiol..

[120] Zhang Q., Shi C., Huang J., Jia K., Chen X., Liu H. (2009). Rapid diagnosis of turbot reddish body iridovirus in turbot using the loop-mediated isothermal amplification method. J. Virol. Methods.

[121] Sung C.H., Chi S.C., Huang K.C., Lu J.K. (2010). Rapid Detection of Grouper Iridovirus by Loop-Mediated Isothermal Amplification. J. Mar. Sci. Technol..

[122] Li Q., Yue Z., Liu H., Liang C., Zheng X., Zhao Y., Chen X., Xiao X., Chen C. (2010). Development and evaluation of a loop-mediated isothermal amplification assay for rapid detection of lymphocystis disease virus. J. Virol. Methods.

[123] Subramaniam K., Shariff M., Omar A.R., Hair-Bejo M., Ong B.L. (2014). Use of Acridine Orange to Visually Improve the Loop-mediated Isothermal Amplification for Detection of Infectious Spleen and Kidney Necrosis Virus. Fish Pathol..

[124] Whittington R.J., Steiner K.A. (1993). Epizootic haematopoietic necrosis virus (EHNV): Improved ELISA for detection in fish tissues and cell cultures and an efficient method for release of antigen from tissues. J. Virol. Methods.

[125] Li P., Zhou L., Wei J., Yu Y., Yang M., Wei S., Qin Q. (2016). Development and characterization of aptamer-based enzyme-linked apta-sorbent assay for the detection of Singapore grouper iridovirus infection. J. Appl. Microbiol..

[126] Zhang X., Zhang Z., Li J., Huang X., Wei J., Yang J., Guan L., Wen X., Wang S., Qin Q. (2022). A Novel Sandwich ELASA Based on Aptamer for Detection of Largemouth Bass Virus (LMBV). Viruses.

[127] Kwon S.R., Nishizawa T., Takami I., Yoshimizu M. (2010). Antibody detection against red sea bream iridovirus (RSIV) in yellowtail *Seriola quinqueradiata* using ELISA. Fish Pathol..

[128] Kim T.-J., Jang E.-J., Kim J.-S., Lee J.-I. (2006). Iridovirus infection of cultured juvenile flounder (*Paralichthys olivaceus*) in nursery. Korean J. Vet. Res..

[129] Eto N., Yamada K., Koga A., Shirahata S., Murakami H. (1991). Establishment and characterization of monoclonal antibodies against Chuzan virus K-47. Cytotechnology.

[130] Matsuyama T., Sano N., Takano T., Sakai T., Yasuike M., Fujiwara A., Kawato Y., Kurita J., Yoshida K., Shimada Y. (2018). Antibody profiling using a recombinant protein-based multiplex ELISA array accelerates recombinant vaccine development: Case study on red sea bream iridovirus as a reverse vaccinology model. Vaccine.

[131] Zhang M., Yang J.X., Lin X.M., Zhu C.H., He J.Q., Liu H., Lin T.L. (2010). A double antibody sandwich enzyme-linked immunosorbent assay for detection of soft-shelled turtle iridovirus antigens. J. Virol. Methods.

[132] Jin Y., Bergmann S.M., Mai Q., Yang Y., Liu W., Sun D., Chen Y., Yu Y., Liu Y., Cai W. (2022). Simultaneous isolation and identification of largemouth bass virus and rhabdovirus from moribund largemouth bass (*Micropterus salmoides*). Viruses.

[133] Zhang Y., Zhang C., Zhang Z., Sun W., Zhang X., Liu X. (2023). Analysis of the transcriptomic profiles of Mandarin fish (*Siniperca chuatsi*) infected with red sea bream iridovirus (RSIV). Microb. Pathog..

[134] Chuang H.-C., Chu T.-W., Cheng A.-C., Chen N.-Y., Lai Y.-S. (2022). Iridovirus isolated from marine giant sea perch causes infection in freshwater ornamental fish. Aquaculture.

[135] Whittington L.A.R.J. (1996). Pathology of epizootic haematopoietic necrosis virus (EHNV) infection in rainbow trout (*Oncorhynchus mykiss* Walbaum) and redfin perch (*Perca fluviatilis* L.). J. Comp. Pathol..

[136] Jensen B.B., Ersbøll A.K., Ariel E. (2009). Susceptibility of pike *Esox lucius* to a panel of Ranavirus isolates. Dis. Aquat. Org..

[137] Vaniksampanna A., Manajit O., Senapin S., Kamsamarn S., Wangman P., Longyant S., Chaivisuthangkura P. (2023). Generation of monoclonal antibodies against heterologously expressed major capsid protein of infectious spleen and kidney necrosis virus (ISKNV). Aquaculture.

[138] Lin H.Y., Liou C.J., Cheng Y.H., Hsu H.C., Yiu J.C., Chiou P.P., Lai Y.S. (2014). Development and application of a monoclonal antibody against grouper iridovirus (GIV) major capsid protein. J. Virol. Methods.

[139] Nakajima K., Maeno Y., Fukudome M., Fukuda Y., Tanaka S., Matsuoka S., Sorimachi M. (1995). Immunofluorescence test for the rapid diagnosis of red sea bream iridovirus infection using monoclonal antibody. Fish Pathol..

[140] Bigarré L., Cabon J., Baud M., Pozet F., Castric J. (2008). Ranaviruses associated with high mortalities in catfish in France. Bull.-Eur. Assoc. Fish Pathol..

[141] Huang C., Zhang X., Gin K.Y.H., Qin Q.W. (2004). In situ hybridization of a marine fish virus, Singapore grouper iridovirus with a nucleic acid probe of major capsid protein. J. Virol. Methods.

[142] Glenn J.A., Emmenegger E.J., Grady C.A., Roon S.R., Gregg J.L., Conway C.M., Winton J.R., Hershberger P.K. (2012). Kinetics of Viral Load and Erythrocytic Inclusion Body Formation in Pacific Herring Artificially Infected with Erythrocytic Necrosis Virus. J. Aquat. Anim. Health.

[143] Davies A.J., Curtis L., Grutter A.S., Smit N.J. (2009). Suspected viral erythrocytic necrosis (VEN) in a juvenile blackbar triggerfish, Rhinecanthus aculeatus, from Lizard Island, Great Barrier Reef, Australia. Mar. Biodivers. Rec..

[144] Qin Q.W., Gin K.Y.-H., Lee L.Y., Gedaria A.I., Zhang S. (2005). Development of a flow cytometry based method for rapid and sensitive detection of a novel marine fish iridovirus in cell culture. J. Virol. Methods.

[145] Cho H.S., Kim T.J. (2007). Comparison of surface plasmon resonance imaging and enzyme-linked immunosorbent assay for the detection of antibodies against iridovirus in rock bream (*Oplegnathus fasciatus*). J. Vet. Diagn. Investig..

[146] Li P., Yan Y., Wei S., Wei J., Gao R., Huang X., Huang Y., Jiang G., Qin Q. (2014). Isolation and characterization of a new class of DNA aptamers specific binding to Singapore grouper iridovirus (SGIV) with antiviral activities. Virus Res..

[147] Jeong J.B., Cho H.J., Jun L.J., Hong S.H., Chung J.-K., Do Jeong H. (2008). Transmission of iridovirus from freshwater ornamental fish (pearl gourami) to marine fish (rock bream). Dis. Aquat. Org..

[148] Hanson L.A., Petrie-Hanson L., Meals K.O., Chinchar V.G., Rudis M. (2001). Persistence of largemouth bass virus infection in a northern Mississippi reservoir after a die-off. J. Aquat. Anim. Health.

[149] Choi S.K., Kwon S.R., Nam Y.K., Kim S.K., Kim K.H. (2006). Organ distribution of red sea bream iridovirus (RSIV) DNA in asymptomatic yearling and fingerling rock bream (*Oplegnathus fasciatus*) and effects of water temperature on transition of RSIV into acute phase. Aquaculture.

[150] Whittington R., Reddacliff G. (1995). Influence of environmental temperature on experimental infection of redfin perch (*Perca fluviatilis*) and rainbow trout (*Oncorhynchus mykiss*) with epizootic haematopoietic necrosis virus, an Australian iridovirus. Aust. Vet. J..

[151] Kurobe T., MacConnell E., Hudson C., McDowell T., Mardones F., Hedrick R. (2011). Iridovirus infections among Missouri River sturgeon: Initial characterization, transmission, and evidence for establishment of a carrier state. J. Aquat. Anim. Health.

[152] Alcorn S.W., Murray A.L., Pascho R.J. (2002). Effects of rearing temperature on immune functions in sockeye salmon (*Oncorhynchus nerka*). Fish Shellfish Immunol..

[153] Jun L., Jeong J., Kim J., Nam J., Shin K., Kim J., Kang J., Jeong H. (2009). Influence of temperature shifts on the onset and development of red sea bream iridoviral disease in rock bream *Oplegnathus fasciatus*. Dis. Aquat. Org..

[154] Leimbach S., Schütze H., Bergmann S.M. (2014). Susceptibility of European sheatfish *Silurus glanis* to a panel of ranaviruses. J. Appl. Ichthyol..

[155] Watson L.R., Milani A., Hedrick R.P. (1998). Effects of water temperature on experimentally-induced infections of juvenile white sturgeon (*Acipenser transmontanus*) with the white sturgeon iridovirus (WSIV). Aquaculture.

[156] Wolf K. (1988). Fish Viruses and Fish Viral Diseases.

[157] Smith G., Blazer V., Walsh H., Iwanowicz L., Starliper C., Sperry A. The effects of disease-related mortality of young-of-year smallmouth bass on the population characteristics in the Susquehanna River basin, Pennsylvania and potential implications to conservation of black bass diversity. Proceedings of the American Fisheries Society Symposium.

[158] Grant E.C., Philipp D.P., Inendino K.R., Goldberg T.L. (2003). Effects of temperature on the susceptibility of largemouth bass to largemouth bass virus. J. Aquat. Anim. Health.

[159] He J., Zeng K., Weng S., Chan S.-M. (2002). Experimental transmission, pathogenicity and physical–chemical properties of infectious spleen and kidney necrosis virus (ISKNV). Aquaculture.

[160] Georgiadis M.P., Hedrick R.P., Carpenter T.E., Gardner I.A. (2001). Factors influencing transmission, onset and severity of outbreaks due to white sturgeon iridovirus in a commercial hatchery. Aquaculture.

[161] Munang’Andu H., Mutoloki S., Evensen Ø. (2016). Prevention and control of viral diseases in aquaculture. Aquaculture Virology.

[162] Mugimba K.K., Byarugaba D.K., Mutoloki S., Evensen Ø., Munang’andu H.M. (2021). Challenges and solutions to viral diseases of finfish in marine aquaculture. Pathogens.

[163] Drennan J.D., Ireland S., LaPatra S.E., Grabowski L., Carrothers T.K., Cain K.D. (2005). High-density rearing of white sturgeon *Acipenser transmontanus* (Richardson) induces white sturgeon iridovirus disease among asymptomatic carriers. Aquac. Res..

[164] Drennan J.D. (2006). Studies on Transmission, Diagnostics, and Immunity to White Sturgeon Iridovirus (WSIV).

[165] Inendino K.R., Grant E.C., Philipp D.P., Goldberg T.L. (2005). Effects of factors related to water quality and population density on the sensitivity of juvenile largemouth bass to mortality induced by viral infection. J. Aquat. Anim. Health.

[166] Min J.G., Jeong Y.J., Jeong M.A., Kim J.-O., Hwang J.Y., Kwon M.-G., Kim K.I. (2021). Experimental transmission of red sea bream iridovirus (RSIV) between rock bream (*Oplegnathus fasciatus*) and rockfish (*Sebastes schlegelii*). J. Fish Pathol..

[167] Essbauer S., Ahne W. (2001). Viruses of Lower Vertebrates. J. Vet. Med. Ser. B.

[168] Macmillan J.R., Mulcahy D., Landolt M.L. (1989). Cytopathology and Coagulopathy Associated with Viral Erythrocytic Necrosis in Chum Salmon. J. Aquat. Anim. Health.

[169] Smail D.A. (1982). Viral erythrocytic necrosis in fish: A review. Proc. R. Soc. Edinb..

[170] Drennan J.D., Lapatra S.E., Samson C.A., Ireland S., Eversman K.F., Cain K.D. (2007). Evaluation of lethal and non-lethal sampling methods for the detection of white sturgeon iridovirus infection in white sturgeon, *Acipenser transmontanus* (Richardson). J. Fish Dis..

[171] Georgiadis M.P., Hedrick R.P., Johnson W.O., Yun S., Gardner I.A. (2000). Risk factors for outbreaks of disease attributable to white sturgeon iridovirus and white sturgeon herpesvirus-2 at a commercial sturgeon farm. Am. J. Vet. Res..

[172] Hedrick R., McDowell T., Groff J., Yun S., Wingfield W. (1992). Isolation and some properties of an iridovirus-like agent from white sturgeon *Acipenser transmontanus*. Dis. Aquat. Org..

[173] LaPatra S., Groff J., Jones G., Munn B., Patterson T., Holt R., Hauck A., Hedrick R. (1994). Occurrence of white sturgeon iridovirus infections among cultured white sturgeon in the Pacific Northwest. Aquaculture.

[174] Schramm Jr H.L., Walters A.R., Grizzle J.M., Beck B.H., Hanson L.A., Rees S.B. (2006). Effects of live-well conditions on mortality and largemouth bass virus prevalence in largemouth bass caught during summer tournaments. N. Am. J. Fish. Manag..

[175] Fusianto C., Hick P.M., Becker J.A. (2019). Stability of Infectious spleen and kidney necrosis virus and susceptibility to physical and chemical disinfectants. Aquaculture.

[176] World Organisation for Animal Health Chapter 10.8 Infection with Red Sea Bream. Aquatic Manual. https://www.woah.org/en/disease/red-sea-bream-iridoviral-disease/.

[177] Munang’Andu H.M., Mutoloki S., Evensen Ø. (2014). Non-replicating vaccines. Fish Vaccination.

[178] Nakajima K., Maeno Y., Honda A., Yokoyama K., Tooriyama T., Manabe S. (1999). Effectiveness of a vaccine against red sea bream iridoviral disease in a field trial test. Dis. Aquat. Org..

[179] Dhar A.K., Manna S.K., Thomas Allnutt F. (2014). Viral vaccines for farmed finfish. Virusdisease.

[180] Zhang J., Fu X., Zhang Y., Zhu W., Zhou Y., Yuan G., Liu X., Ai T., Zeng L., Su J. (2019). Chitosan and anisodamine improve the immune efficacy of inactivated infectious spleen and kidney necrosis virus vaccine in *Siniperca chuatsi*. Fish Shellfish Immunol..

[181] Zhao Z., Xiong Y., Zhang C., Jia Y.-J., Qiu D.-K., Wang G.-X., Zhu B. (2020). Optimization of the efficacy of a SWCNTs-based subunit vaccine against infectious spleen and kidney necrosis virus in mandarin fish. Fish Shellfish Immunol..

[182] Li N., Fu X., Guo H., Lin Q., Liu L., Zhang D., Fang X., Wu S. (2015). Protein encoded by ORF093 is an effective vaccine candidate for infectious spleen and kidney necrosis virus in Chinese perch *Siniperca chuatsi*. Fish Shellfish Immunol..

[183] Dong C., Xiong X., Luo Y., Weng S., Wang Q., He J. (2013). Efficacy of a formalin-killed cell vaccine against infectious spleen and kidney necrosis virus (ISKNV) and immunoproteomic analysis of its major immunogenic proteins. Vet. Microbiol..

[184] Fu X., Li N., Lin Q., Guo H., Liu L., Huang Z., Wu S. (2015). Early protein ORF086 is an effective vaccine candidate for infectious spleen and kidney necrosis virus in mandarin fish *Siniperca chuatsi*. Fish Shellfish Immunol..

[185] Zhao Z., Li Y., Chen G., Zhang C., Wang G.X., Zhu B. (2022). Protective immunity against infectious spleen and kidney necrosis virus induced by mannose modified subunit vaccine with carbon nanotubes in mandarin fish. Aquac. Res..

[186] Caipang C.M.A., Takano T., Hirono I., Aoki T. (2006). Genetic vaccines protect red seabream, *Pagrus major*, upon challenge with red seabream iridovirus (RSIV). Fish Shellfish Immunol..

[187] Tamaru Y., Ohtsuka M., Kato K., Manabe S., Kuroda K., Sanada M., Ueda M. (2006). Application of the arming system for the expression of the 380R antigen from red sea bream iridovirus (RSIV) on the surface of yeast cells: A first step for the development of an oral vaccine. Biotechnol. Prog..

[188] Fan T., Hu X., Wang L., Geng X., Jiang G., Yang X., Yu M. (2012). Development of an inactivated iridovirus vaccine against turbot viral reddish body syndrome. J. Ocean. Univ. China.

[189] Zheng F., Liu H., Sun X., Zhang Y., Zhang B., Teng Z., Hou Y., Wang B. (2016). Development of oral DNA vaccine based on chitosan nanoparticles for the immunization against reddish body iridovirus in turbots (*Scophthalmus maximus*). Aquaculture.

[190] Zheng F., Liu H., Sun X., Qin X., Xu Z., Wang B. (2017). Construction and expression of DNA vaccine against reddish body iridovirus and evaluation of immune efficacy in turbot (*Scophthalmus maximus*). Aquac. Res..

[191] Robinson N.A., Robledo D., Sveen L., Daniels R.R., Krasnov A., Coates A., Jin Y.H., Barrett L.T., Lillehammer M., Kettunen A.H. (2023). Applying genetic technologies to combat infectious diseases in aquaculture. Rev. Aquac..

[192] Jung M.-H., Nikapitiya C., Jung S.-J. (2018). DNA vaccine encoding myristoylated membrane protein (MMP) of rock bream iridovirus (RBIV) induces protective immunity in rock bream (*Oplegnathus fasciatus*). Vaccine.

[193] Zhang M., Hu Y.-H., Xiao Z.-Z., Sun Y., Sun L. (2012). Construction and analysis of experimental DNA vaccines against megalocytivirus. Fish Shellfish Immunol..

[194] Zhu M., Shen Z., Gu Y., Tong X., Zhang Y., Pan J., Feng Y., Hu X., Wang Y., Cao G. (2023). A recombinant baculovirus vector vaccine (BacMCP) against the infectious spleen and kidney necrosis virus (ISKNV). J. Fish Dis..

[195] Ou-Yang Z., Wang P., Huang X., Cai J., Huang Y., Wei S., Ji H., Wei J., Zhou Y., Qin Q. (2012). Immunogenicity and protective effects of inactivated Singapore grouper iridovirus (SGIV) vaccines in orange-spotted grouper, *Epinephelus coioides*. Dev. Comp. Immunol..

[196] Ou-Yang Z., Wang P., Huang Y., Huang X., Wan Q., Zhou S., Wei J., Zhou Y., Qin Q. (2012). Selection and identification of Singapore grouper iridovirus vaccine candidate antigens using bioinformatics and DNA vaccination. Vet. Immunol. Immunopathol..

[197] Yu N.-T., Zheng X.-b., Liu Z.-X. (2019). Protective immunity induced by DNA vaccine encoding viral membrane protein against SGIV infection in grouper. Fish Shellfish Immunol..

[198] Liu H.-I., Chiou P.P., Gong H.-Y., Chou H.-Y. (2015). Cloning of the major capsid protein (MCP) of grouper iridovirus of Taiwan (TGIV) and preliminary evaluation of a recombinant MCP vaccine against TGIV. Int. J. Mol. Sci..

[199] Zheng F.-R., Sun X.-Q., Liu H.-Z., Zhang J.-X. (2006). Study on the distribution and expression of a DNA vaccine against lymphocystis disease virus in Japanese flounder (*Paralichthys olivaceus*). Aquaculture.

[200] Tian J.-Y., Sun X.-Q., Chen X.-G. (2008). Formation and oral administration of alginate microspheres loaded with pDNA coding for lymphocystis disease virus (LCDV) to Japanese flounder. Fish Shellfish Immunol..

